# Design and Development of Microcontroller-Based Clinical Chemistry Analyser for Measurement of Various Blood Biochemistry Parameters

**DOI:** 10.1155/JAMMC.2005.223

**Published:** 2005

**Authors:** S. R. Taneja, R. C. Gupta, Jagdish Kumar, K. K. Thariyan, Sanjeev Verma

**Affiliations:** 1 Central Scientific Instruments Organisation Sector-30 Chandigarh 160 030 India

## Abstract

Clinical chemistry analyser is a high-performance microcontroller-based photometric biochemical analyser to measure various blood biochemical parameters such as blood glucose, urea, protein, bilirubin, and so forth, and also to measure and observe enzyme growth occurred while performing the other biochemical tests such as ALT (alkaline amino transferase), amylase, AST (aspartate amino transferase), and so forth. These tests are of great significance in biochemistry and used for diagnostic purposes and classifying various disorders and diseases such as diabetes, liver malfunctioning, renal diseases, and so forth. An inexpensive clinical chemistry analyser developed by the authors is described in this paper. This is an open system in which any reagent kit available in the market can be used. The system is based on the principle of absorbance transmittance photometry. System design is based around 80C31 microcontroller with RAM, EPROM, and peripheral interface devices. The developed system incorporates light source, an optical module, interference filters of various wave lengths, peltier device for maintaining required temperature of the mixture in flow cell, peristaltic pump for sample aspiration, graphic LCD display for displaying blood parameters, patients test results and kinetic test graph, 40 columns mini thermal printer, and also 32-key keyboard for executing various functions. The lab tests conducted on the instrument include versatility of the analyzer, flexibility of the software, and treatment of sample. The prototype was tested and evaluated over 1000 blood samples successfully for seventeen blood parameters. Evaluation was carried out at Government Medical College and Hospital, the Department of Biochemistry. The test results were found to be comparable with other standard instruments.


Journal of Automated Methods & Management in Chemistry, **2005** (2005), no. 4, 223–229
© 2005 Hindawi Publishing Corporation

### Design and Development of Microcontroller-Based Clinical Chemistry Analyser for Measurement of Various Blood Biochemistry Parameters

S. R. Taneja, R. C. Gupta, Jagdish Kumar, K. K. Thariyan, and Sanjeev Verma

*Central Scientific Instruments Organisation, Sector-30, Chandigarh-160 030, India*

*Received 13 December 2004; Accepted 21 June 2005*

Clinical chemistry analyser is a high-performance microcontroller-based photometric biochemical analyser to measure various blood biochemical parameters such as blood glucose, urea, protein, bilirubin, and so forth, and also to measure and observe enzyme growth occurred while performing the other biochemical tests such as ALT (alkaline amino transferase), amylase, AST (aspartate amino transferase), and so forth. These tests are of great significance in biochemistry and used for diagnostic purposes and classifying various disorders and diseases such as diabetes, liver malfunctioning, renal diseases, and so forth. An inexpensive clinical chemistry analyser developed by the authors is described in this paper. This is an open system in which any reagent kit available in the market can be used. The system is based on the principle of absorbance transmittance photometry. System design is based around 80C31 microcontroller with RAM, EPROM, and peripheral interface devices. The developed system incorporates light source, an optical module, interference filters of various wave lengths, peltier device for maintaining required temperature of the mixture in flow cell, peristaltic pump for sample aspiration, graphic LCD display for displaying blood parameters, patients test results and kinetic test graph, 40 columns mini thermal printer, and also 32-key keyboard for executing various functions. The lab tests conducted on the instrument include versatility of the analyzer, flexibility of the software, and treatment of sample. The prototype was tested and evaluated over 1000 blood samples successfully for seventeen blood parameters. Evaluation was carried out at Government Medical College and Hospital, the Department of Biochemistry. The test results were found to be comparable with other standard instruments.

### 1. INTRODUCTION

In order to measure the progress of an enzymatic reaction and to measure the total change in the concentration of the reactant/substrate, various techniques [1] such as spectrophotometric, polarometric, amperometric, electrochemical, coulometric, polarography, radiochemical, and fluorescence are available. Instrument developed works on the principle of *absorbance transmittance photometry*. It is a high-performance, microcontroller-based, photometric biochemical analyser to measure various blood biochemical parameters such as blood glucose, urea, protein, bilirubin, and so forth, and also to measure and observe enzyme growth occurred while performing the other biochemical tests such as ALT (alkaline amino transferase), amylase, AST (aspartate amino transferase), and so forth. The biochemical tests are very important as they are associated with various disorders and diseases such as diabetes, renal diseases, liver malfunctions, and other metabolic derangements. The quantisation of these parameters is helpful in classifying such diseases, and under appropriate circumstances, results are used for diagnostic purposes.

In recent years, automation in clinical chemistry has progressed with a change from rigid to very flexible instruments. Automation of clinical instruments has brought about a revolution in the field of medical instrumentation. It has reduced the load on clinical laboratories to a great extent by reducing the time taken in the test and minimizing the involvement of laboratory staff. Instrument developed is classified as semiautomated analyser [2] and has advantages of precision and accuracy. These systems are used in hospitals to test various blood biochemical parameters. All primary health centres, community health centres, and district hospitals are the potential users of this machine.

### 2. MATERIALS AND METHODS

### 2.1. The instrument

*(i) Design principle*

The instrument is designed using the principle of *absorbance transmittance photometry*. According to Lambert and Beer's

Correspondence and reprint requests to R. C. Gupta, Central Scientific Instruments Organisation, Sector-30, Chandigarh – 160 030, India; E-mail: rcg_csio@yahoo.co.in.

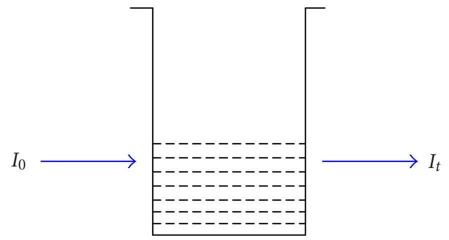

FIGURE 1: Schematic of Lambert and Beer's law.

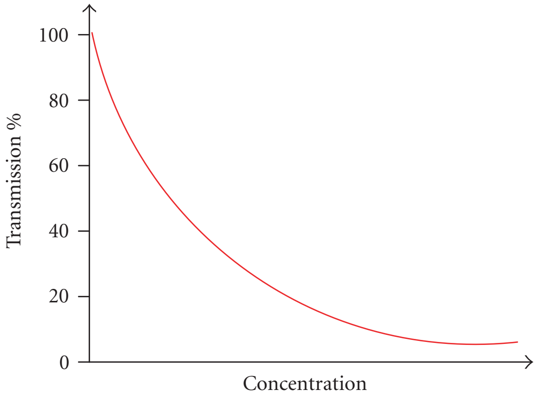

FIGURE 2: Relation between percent transmission and concentration.

law [3], when monochromatic light is passed through coloured solution, the intensity of the transmitted light decreases exponentially with the increase in concentration of the absorbing substance. The value of absorption of light energy is dependent on the number of molecules present in absorbing material and the thickness of the medium. Thus, intensity of light energy leaving the absorbing substance is used as an indication of concentration of that particular substance.

As shown in Figures 1 and 2, if $I_0$ is the intensity of incident light in coloured solution and $I_t$ is the transmitted light, then according to this law

$$I_t = I_0 e^{-kct}, \tag{1}$$

and transmission

$$T = \frac{I_t}{I_0} = e^{-kct}, \tag{2}$$

or

$$\log_e T = -kct, \tag{3}$$

or

$$\log_e \left(\frac{1}{T}\right) = kct, \tag{4}$$

where $c$ is the concentration of absorbing material, $t$ thickness of the light path, and $k$ absorption constant.

The quantity $(-\log T)$ or $\log(1/T)$ is termed as extinction E/OD or the absorbance:

$$A = \log\left(\frac{1}{T}\right) = \log 100/(\%\text{transmission}), \quad A = 2 - \log(\%\text{transmission}). \tag{5}$$

Therefore $A = kct$.

If $t$ is constant, then $A \alpha C$.

In this system, the basic requirement is to measure optical density/absorbance and then concentration of the test parameter under run accurately.

*(ii) Microcontroller-based hardware*

Figure 3 represents the basic modules of the system; a light source, an optical module, a filter wheel, a quartz cuvette with reaction mixture, a photo detector, and signal processing circuitry based on microcontroller. Overall system design is shown in Figure 4. The block diagram can be subdivided into the following major parts:

(i) the microcontroller and memories,
(ii) peripherals and interfaces.

System design is based around 80C31 microcontroller [4] connected through address bus, data bus, and control bus to the 64 kbytes of EPROM 27C512 for monitor and control program, 24 kbytes of RAM with battery backup for temporary data storage, 24-hour results storage capacity, and peripheral I/O devices 8255 s are used for interfacing 32-key keyboard, 12-bit A/D converter, 40 column thermal mini printer, and 30 characters × 8 lines alphanumeric/graphic LCD display. Alphanumeric keyboard contains various function keys, numeric keys and aspirate and RESET key for selecting various functions of the system and parameter analysis. The LCD is used for displaying date and time by real-time clock, various menus, parameters and data entered from keyboard as well as patient's results and kinetic graphs as required in some test parameters. 12-bit A/D converter converts analog signal from photo detector and preamplifier into digital form. 40 column thermal mini printer is used for hard copy of the parameters stored, patient test results, collection report, and kinetic graphs as displayed on the LCD. These graphs facilitate authenticity of the test results.

*(iii) Peltier-based temperature controller*

System developed is used to determine both enzyme activity and substrate concentration in biological fluids at different temperatures 25 °C, 30 °C, and 37 °C by initial rates using fixed time, end point, and kinetic methods. System allows selecting any required temperature and maintains the temperature of flow cell at that selected temperature because enzymes are relatively fragile substances which have a tendency to undergo inactivation or denaturation [1]. So to get the proper enzymatic rate and for increasing the stability they must be properly handled while conducting the test and must be kept at required temperatures. Temperature sensor LM335

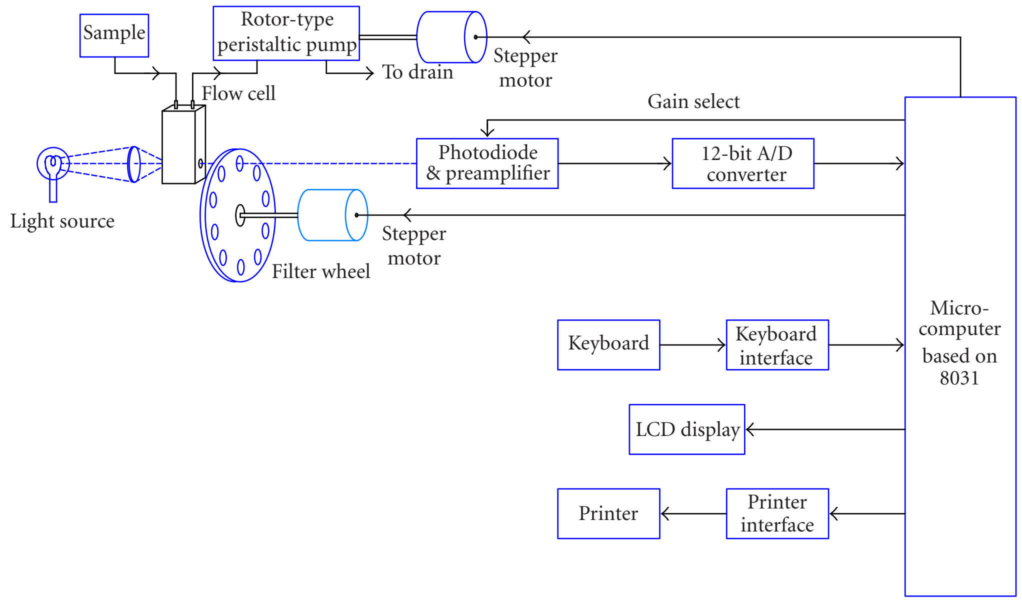

FIGURE 3: Block diagram of clinical chemistry analyser.

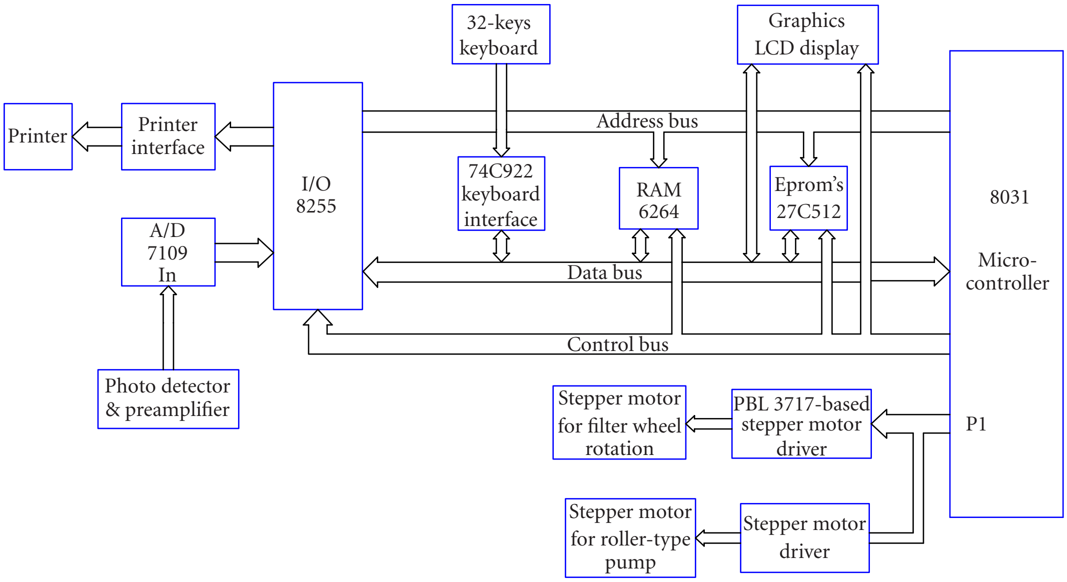

FIGURE 4: Microcontroller-based hardware design of clinical chemistry analyser.

and peltier device are used to provide and maintain the required temperature for the samples in flow cell. Peltier works in both directions for cooling and heating. This effect is used to control the temperature of the sample.

*(iv) Peristaltic pump/aspiration system*

Port1 of 8031 microcontroller is interfaced with a stepper motor through a driver hardware that drives the roller type of peristaltic pump which generates the required sequence of pulses for the motor driving hardware. The driver hardware enhances the level of voltage for pulse sequences required for the stepper motor. Roller type peristaltic pump used in the system is aspirating the required volume of reagents/samples and for washing the flow cell. This pump can be calibrated to aspirate the required quantity of water, reagent, and samples.

*(v) Optical module and filter wheel assembly*

Optical module consists of a light source with reflector, condenser system, collimating objectives, flow cell, filter wheel assembly, and photodiode. Halogen lamp is used as a light

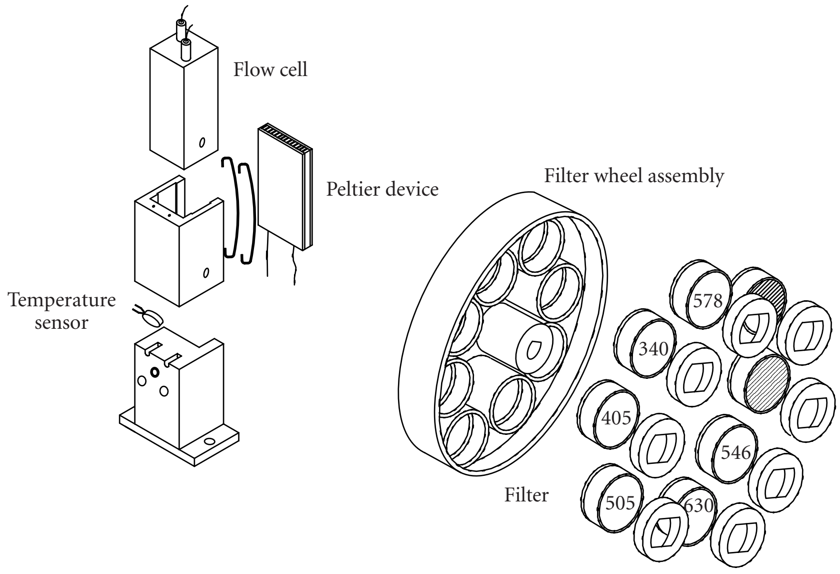

Figure 5: Isometric view of filter wheel assembly and Peltier device.

source. A constant current power supply is used to power the lamp to reduce the fluctuations in the light. All optical components have been designed with quartz glass to have good transmission in UV region at 340 nm. Keeping in view the low response of photo detector in UV (340 nm), all the optical components have been provided with enhanced antireflection coating in the UV region. In the opto-mechanical assembly, special care has been taken in the design so that each component is properly aligned with respect to optical axis. To get the required wavelength of light to be passed, 6 interference filters of different wavelengths such as 340 nm, 405 nm, 505 nm, 546 nm, 578 nm, and 630 nm, from UV region to visible region spectrum (300 nm to 700 nm), have been mounted on the filter wheel. These filters are selected automatically depending on the test performed. When the filter of required wavelength is selected, the corresponding gain is selected automatically. The filter wheel is driven by a stepper motor, which is interfaced with the port of microcontroller through driver circuit. Pulses are generated according to required sequence to rotate the motor at required angle, which brings the filter in front of photo detector. The diagram of filter wheel assembly is shown in Figure 5.

### 2.2. Methods

*(i) Signal processing*

Advances in electronics and microcontroller technology have played a central role in signal processing [5]. Computers are included in automated analysis for data acquisition and processing of analytical data. Output of photodiode and preamplifier is a voltage which varies directly with the light which is passed through the flow cell and selected wavelength filter. Preamplifier gain is selected automatically as per the selected test parameter and filter. Output of preamplifier is converted into digital value by a 12-bit analog to digital converter. Microcontroller performs calculations on these digital values according to the appropriate calculation algorithms developed [6].

### 2.3. Methods for calculation

The instrument developed works in four different modes such as concentration (end point), kinetic mode, fixed-time mode, and absorbance mode. For measurement of concentration in different modes, different formulas are used as shown below.

*(1) Concentration (end point) mode:*

$$\text{Concentration of sample} = \text{Abs. Sample} \times \frac{\text{Conc. of Standard}}{\text{Abs. of Standard}} \tag{6}$$

or

$$\text{Concentration of sample} = \text{Abs. Sample} \times F. \tag{7}$$

*(2) Kinetic mode:*

$$\begin{aligned}\text{Concentration } (U/L) &= \Delta\,\text{Abs./Min} \times \frac{\text{Conc. of Standard}}{\text{Abs/min of Standard}} \quad \text{or} \\ &= \Delta\,\text{Abs / Min} \times F.\end{aligned} \tag{8}$$

*(3) Fixed-time mode:*

$$\begin{aligned}\text{Concentration of sample} &= \Delta\,\text{Abs.} \times \frac{\text{Conc. of Standard}}{\Delta\,\text{Abs. of Standard}} \quad \text{or} \\ &= \Delta\,\text{Abs.} \times F,\end{aligned} \tag{9}$$

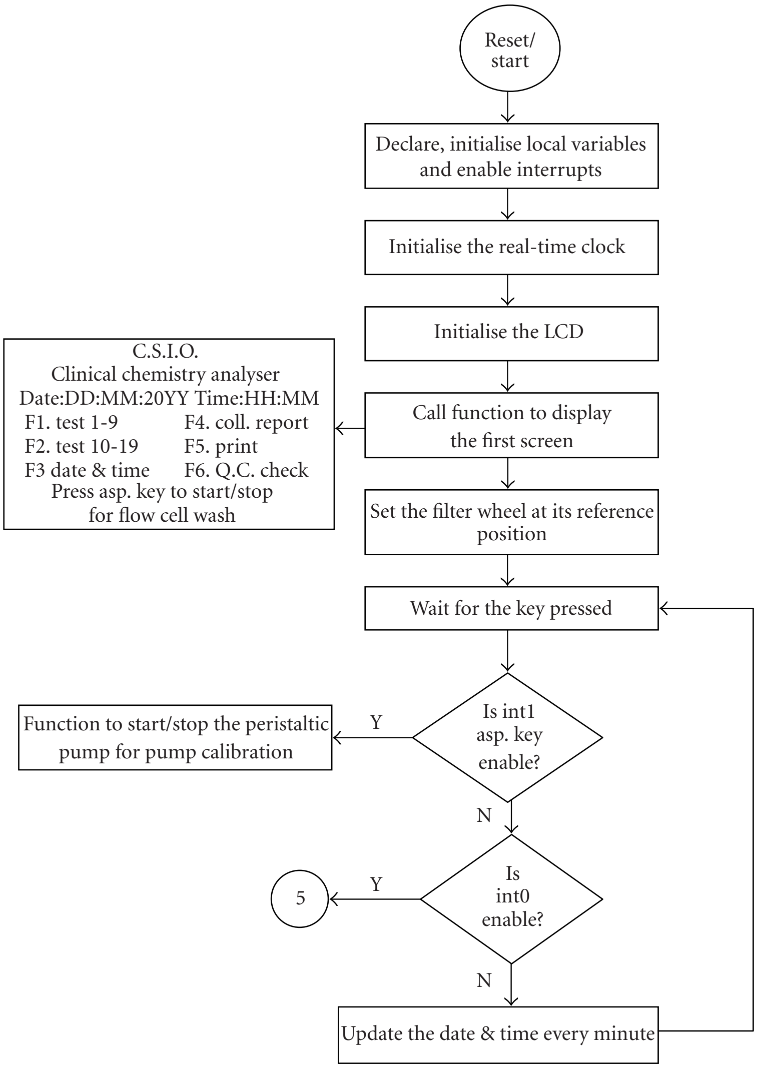

Figure 6: Flow diagram of system software.

where

$$F = \frac{\text{T.V.} \times 10^6}{\text{S.V.} \times \text{Absorptivity} \times \text{P}}, \qquad (10)$$

T.V. is total reaction volume, S.V. is sample volume, P is path length in cm, Abs. is absorbance, Conc. is concentration, and Min. is minute.

### *2.4. System software*

The layout of the steps followed in the development of the software of the instrument has been provided in the flow chart shown in Figures 6 and 7. On the basis of this flow diagram, system software has been developed using "C" cross compiler [7, 8] for Intel 8031 microcontroller in modular form. System software is menu driven and user friendly. Many advance features have been incorporated in the software for fast and robust operation. The main program calls the subfunctions and executes them accordingly. System software is stored in the system EPROM.

### 3. RESULTS AND DISCUSSION

In this system, the programming, reading, and reporting operations are easy and user friendly. The instrument is provided with a keyboard which facilitates quick change from one function to another and setting of parameters which can be monitored on LCD with both alphanumeric as well as graphics capabilities without going through complex sequential operations. Printer in the system is used for test reports of the patients. The system was clinically evaluated successfully over 1000 blood samples at the Department of Biochemistry, Government Medical College & Hospital, Sector-32, Chandigarh. 17 blood parameters have been analysed and results have been found to be satisfactory.

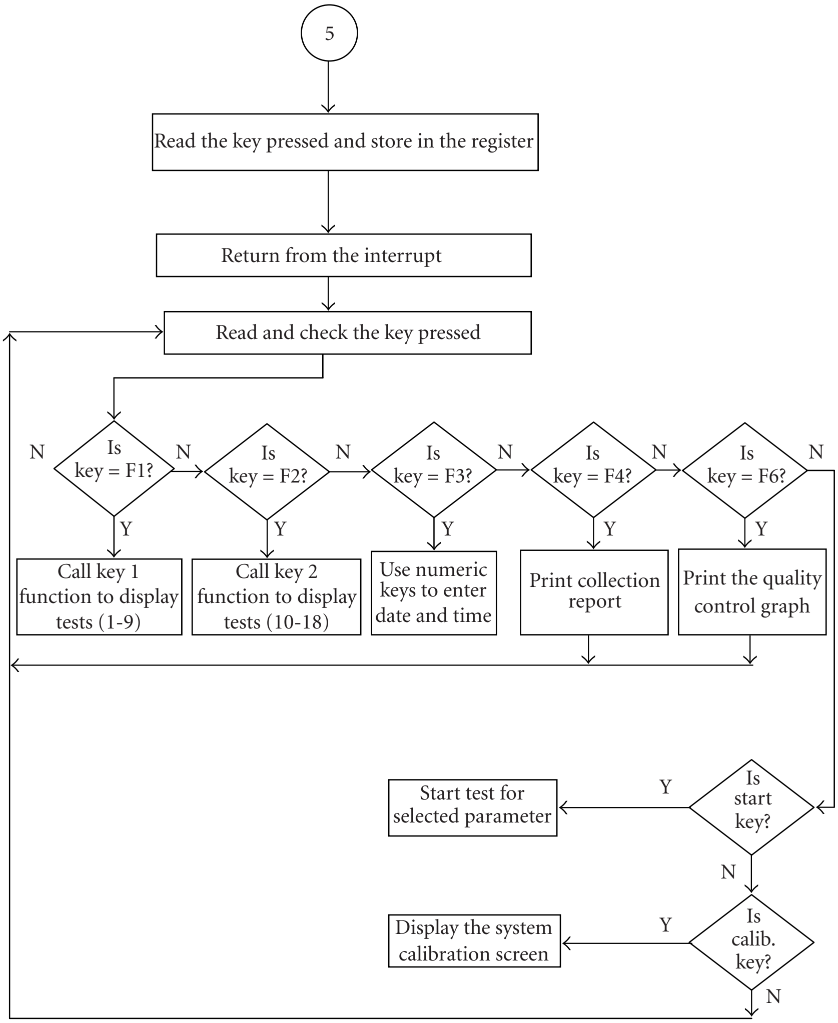

Figure 7: Flow diagram of system software.

### 4. CONCLUSION

The instrument developed is universally useful for small clinical laboratories, big hospitals, and nursing homes for qualitative analysis of blood. The instrument is capable of handling a reasonable amount of workload. The work organization of the instrument is most efficient when batches of tests are analyzed together. In this way, a discretionary approach can be achieved without affecting the performance of instrument. End point samples can be given priority at any time during a routine run. Menu-driven system software provides a user-friendly environment with many attractive features for easy operation requiring minimum training of the operator. The technology of this system has been transferred to Indian industry [10].

### ACKNOWLEDGMENTS

The authors are deeply grateful to Dr Pawan Kapur, Director, Dr P. K. Jain and Mr P. K. Goel, Scientists of CSIO, Chandigarh, and Dr Jasbinder Kaur, Acting Head, Department of Biochemistry, GMCH-32, Chandigarh, for providing the necessary facilities and help during the progress of work and clinical evaluation of the system. The project was sponsored by the Department of Science and Technology, New Delhi, India.

### REFERENCES

[1] G. G. Guilbault, *Study of Handbook of Enzymatic Methods of Analysis*, Marcel Dekker, New York, NY, USA, 1976.

[2] R. Haeckel, "General principles for the classification of analysers," *Journal of Automated Methods and Management in Chemistry*, vol. 10, no. 4, pp. 164–166, 1988.

[3] D. C. Harris, *Quantitative Chemical Analysis*, W. H. Freeman & Company, New York, NY, USA, 4th edition, 1995.

[4] I. S. MacKenzie, *The 8051 Microcontroller*, Prentice-Hall, Englewood Cliffs, NJ, USA, 2nd edition, 1995.

[5] P. A. Bonini, F. Ceriotti, and C. Franzini, "Selectivity and random-access in automatic analysers," *Journal of Automated Methods and Management in Chemistry*, vol. 10, no. 4, pp. 167–170, 1988.

[6] T. W. Schultz, *C and the 8051, vol. I: Hardware, Modular Programming & Multitasking*, Prentice-Hall, Englewood Cliffs, NJ, USA, 2nd edition, 1998.
[7] User Manual 8051 C Complier Programming Guide for Intel's 8051 Microcontroller Family, IAR SYSTEMS.
[8] User Manual winIDEA$^{TM}$ Version 9.0, Integrated Development Environment, Software User's Guide, iSYSTEM.
[9] P. A. Bonini, E. Callioni, F. Ceriotti, et al., "Multicentre evaluation of the IL densiscan," *Journal of Applied Mathematics and Stochastic Analysis*, vol. 8, no. 1, pp. 18–22, 1986.
[10] "Technical manual of Clinical Chemistry Analyser developed by CSIO," 2003.

